# Phyto-treatment of tannery industry effluents under combined application of citric acid and chromium-reducing bacterial strain through *Lemna minor* L.: A lab scale study

**DOI:** 10.1016/j.heliyon.2024.e36309

**Published:** 2024-08-13

**Authors:** Rahat Arshad, Arwa Abdulkreem AL-Huqail, Suliman Mohammed Suliman Alghanem, Ibtisam Mohammed Alsudays, Mujahid Farid, Wajiha Sarfraz, Mohsin Abbas, Zaki ul Zaman Asam, Noreen Khalid, Jean Wan Hong Yong, Amany H.A. Abeed

**Affiliations:** a Department of Environmental Sciences, University of Gujrat, Hafiz Hayat Campus, Gujrat, 50700, Pakistan; b Department of Biology, College of Science, Princess Nourah bint Abdulrahman University, P.O.Box 84428, Riyadh, 11671, Saudi Arabia; c Department of Biology, College of Science, Qassim University, Burydah, 52571, Saudi Arabia; d Australia Rivers Institute and School of Environment and Science, Griﬃth University, Nathan, QLD, 4111, Australia; e Department of Botany, Government College Women University, Sialkot, Pakistan; f Department of Biosystems and Technology, Swedish University of Agricultural Sciences, 23456, Alnarp, Sweden; g Department of Botany and Microbiology, Faculty of Science, Assiut University, Assiut, 71516, Egypt

**Keywords:** Chromium toxicity, *Lemna minor*, Remediator, *Staphylococcus aureus* strain K1, Tannery effluents, Citric acid

## Abstract

Contamination of agricultural soils with heavy metals (HMs) poses a significant environmental threat, especially because industrial discharges often irrigate agricultural lands. A prominent source of HM(s) pollution occurs from tannery effluents containing high concentrations of chromium (Cr) in both Cr^3+^ and Cr^6+^ forms along with other toxic materials. Cr is known for its carcinogenic and mutagenic properties in biological systems. Microbe-assisted phytoremediation has emerged as a promising and environmentally friendly approach for detoxifying Cr-contaminated environments. This study aimed to evaluate the performance of citric acid (CA) and a Cr-reducing bacterial strain (*Staphylococcus aureus)* on the phytoextraction potential of *Lemna minor* within a Constructed Wetland System treated with tannery wastewater. Various combinations of tannery wastewater (0, 50, and 100 %), CA (0, 5 and 10 mM), and microbial inoculants were applied to the test plants. The mitigative effects of *Staphylococcus aureus* strain K1 were examined in combination with different concentrations of CA (0, 5, 10 mM). Data on growth and yield attributes highlighted the beneficial effects of bacterial inoculation and CA in ameliorating Cr toxicity in *L. minor*, as evidenced by increased foliar chlorophyll and carotenoid contents, enhanced antioxidant enzyme activities (SOD, POD, APX, CAT), and improved nutrient uptake. Specifically, CA application resulted in an enhancement of Cr ranging from 12% to 15% and 23%–31% in concentration, and 134%–141% and 322%–337% in Cr accumulation, respectively. When combined with the *S. aureus* inoculation treatment, CA application (5 and 10 mM) further increased the concentration and accumulation of Cr in L. *minor*. The enhancement in Cr ranged from 12% to 23% and 27%–41% in concentration, 68%–75%, and 179%–185% in accumulation, respectively. These results demonstrated that *L. minor* is an effective choice for environmentally friendly Cr remediation due to its continued ability to grow in polluted wastewater. This study suggested that microbial-assisted phytoextraction combined with chelating agents such as CA could be a practical and effective approach for remediating tannery effluents.

## 1 Introduction

With the increasing anthropogenic activities, such as those in construction, industries and agriculture, the multi-facets of soil and water pollution are becoming increasingly prominent. Chromium (Cr), a pollutant identified in tannery effluent at elevated levels than other metals, poses significant risks to human health, animals, and plants. In aquatic ecosystems, Cr exists in chromium (III) and chromium (VI) in two predominant oxidation states. The Cr^3+^ is characterized by its lower toxicity, primarily attributed to its propensity for precipitation. Conversely, Cr^6+^, also known as chromate, represents the more hazardous manifestation of chromium. Chromate's potent oxidizing capabilities and enhanced membrane transport efficiency which contribute to its increased toxicity. Therefore, identifying various effective strategies to mitigate heavy metals (HMs) pollution is essential for safeguarding the ecosystem and human liveability [[1], [2], [3], [4], [5], [6]].

Conventional physicochemical and biological methodologies are proven effective in reducing HM(s) toxicity in plants [[3], [6], [7], [8], [9]]. Phytoremediation serves as a mechanism to transform environmentally prevalent toxic metals into less harmful forms [[3], [6], [10], [11]]. Increasing interest in recent years has been reported regarding phytoremediation as a potential approach to alleviate heavy metal and metalloid contamination in many different scenarios. Recently, the widespread adoption of advanced technologies, like constructed wetland system (CWs) for waste water (WW) treatment, has garnered attention due to their comparative advantages over alternative technologies for sustainability [12,[13], [14]]. Aquatic macrophytes can effectively accumulate and remove Cr from various contaminated environments. The uptake of Cr by macrophytes is species dependent and there are some species displaying a high biological ability for the accumulation of this metal [[3], [15]].

From literature review and our surveys, one macrophyte species, *Lemna minor*, was identified for plausible Cr extraction from tannery WW [16]. Uysal (2013) demonstrated that *L. minor* could be harnessed in a continuous flow pond system to simulate conditions similar to those in a wastewater treatment environment. The cell walls of this floating species are known to possesses functional groups involved in the binding of Cr ions. These groups include carboxyl, phosphate, thiol, peptide, and hydroxide [17]. In addition, using chemically synthesized agents, organic acids, amino acids, and other substances can enhance remediation efficiency Farid et al., 2022. Among these, organic acids like citric acid are particularly effective due to their lower chance of desorbing and higher resistance to degradation compared to other chelates. Our previous studies have shown that combining *L. minor* with citric acid (CA) successfully removes Cr from tannery WW. Citric acid can bind with metal ions that form complex molecules that are less toxic and more readily sequestered within the plant's tissues. Metals absorbed by *L. minor* can be contained within distinct cellular compartments, such as vacuoles, thereby diminishing their toxicity. Furthermore, certain metals may undergo metabolic conversions within the plant, leading to their immobilization or detoxification [[6], [18]].

However, remediating HMs from water resources remains a significant challenge, as individual plants alone are insufficient to eliminate the toxicity of HMs in contaminated areas [[3], [6], [14], [19]]. Fortunately, microbes associated with phytoremediation employ various mechanisms that significantly contribute to the remediation of HMs contamination, making them suitable for large-scale implementation [20[21], [22]]. Chromate exhibits active transport across eukaryotic and prokaryotic cell membranes. The involvement of the sulfate uptake pathways in facilitating chromate transport across biological membranes in various bacteria, including *Salmonella typhimurium*, *Escherichia coli* [23], *Pseudomonas fluorescent* [24], *Alcaligenes eutrophus and Staphylococcus aureus* [25], have been reported. The prevalence of the oxyanion form of chromate (CrO_2_Q) is responsible for hindering entrapment by the anionic constituents of the cell membrane, contributing to this phenomenon. It was reported that *S. aureus* capable of Cr reduction can mitigate Cr toxicity; by converting Cr^6+^ into Cr^3+^ within the rhizosphere through the mechanisms of bioaccumulation and biosorption within the tannery effluent [[26], [27]]. This reduces the toxicity of metals in the environment, which may enhance their uptake by *L. minor.*

The rationale behind this methodology originates from the hypothesis that bacteria subjected to WW containing hazardous metals may have undergone plausible biological adaptation and acclimatization to adverse environmental conditions. Additionally, the extreme environments characteristic of metal-rich wastewater hold the potential to yield novel bacterial strains that inherently exhibit resistance to elevated concentrations of toxic metals. This research centers on bacterial strain (i.e. *Staphylococcus aureus* K1) tolerant to Cr within tannery effluent for potential utilization in environmental remediation practices. Moreover, this study also links with our previous study that used *Lemna minor* and citric acid to remove Cr from tannery effluents. The novelty of this experiment lies in harnessing the tolerant bacterial strain living within tannery effluent; with the intrinsic capability to reduce Cr^6+^ to Cr^3+^^;^ alongside an assessment of the capacity of *Lemna minor*, in conjunction with citric acid, to accumulate Cr. Thus, the primary objectives of this study are: (a) to investigate the morpho-physiological characteristics of *L. minor* when subjected to wastewater treatments; (b) to assess the effectiveness and capacity of *L. minor* in the bioaccumulation of Cr using citric acid, both with and without the presence of *Staphylococcus aureus*; (c) to determine the potential of *L. minor*, with or without microbial intervention, in reducing oxidative stress and enhancing antioxidant mechanisms through citric acid treatment.

## 2 Materials and methods

### 2.1 Experimental setup

Fresh L. *minor* plants were harvested from a controlled environment adjacent to a pond and marshy area in Gujrat, while wastewater samples were from the tannery industry, Sambrial. Before transplantation into the CWs, the plants underwent washing with distilled water. The light was provided by a 100W electric bulb for photosynthesis. Submersible pumps were used to ensure continuous aeration of the experimental plants. The roots were submerged in the liquid solution while the rest of the plants remained floating above the water's surface. Throughout the experiment, 1.0 M H_2_SO_4_ was added to the nutrient solution to maintain a conducive pH range of 5.8–6.3. Standard analytical techniques were used to assess the physicochemical characteristics of the tannery effluents, as described in Supplementary Table 1.

### 2.2 Bacterial inoculum with wastewater and citric acid

The bacterial inoculum was prepared using the method described by Zeng et al. [28]. Individual bacterial colonies were cultured in 250 mL of nutrient broth and incubated on a rotary shaker at 37 °C for 24 h. After that, the overnight culture was harvested by centrifugation at 10,000 rpm for 10 min. The supernatant was discarded, and the bacterial pellet obtained was washed with deionized water. Finally, the pellet was resuspended in a normal saline solution (0.85 % NaCl). After a two-week treatment period, the plants underwent exposure to bacterial colonies and various concentrations of WW (0 %, 50 %, and 100 %, along with citric acid (0 mM, 5 mM, and 10 mM). All treatments were administered appropriately with their various inputs weekly.

### 2.3 Treatment levels

Three replicates of each treatment were used in a completely randomized design (CRD). The different treatments were designed with and without microbes as: Control, 0 % WW + OA (5 mM), 0 % WW + OA (10 mM), 50 % WW + OA (0 mM), 50 % WW + OA (5 mM), 50 % WW + OA (10 mM), 100 % WW + OA (0 mM), 100 % WW + OA (5 mM), 100 % WW + OA (10 mM).

### 2.4 Assessment of growth and biochemical traits

After a six-week treatment period, the plants were harvested for morphological and biochemical analysis. Fresh biomass (200 plants per growth parameter) was determined using an analytical weighing scale. Plant material was then dried in an oven at 90 °C until a constant weight was attained to assess dry weight. Fresh leaf samples weighing 0.2 g were weighed using an analytical balance. These samples were then submerged in 10 mL of 80 % acetone and allowed to soak overnight in a controlled environment with minimal light exposure. After maceration, the solution underwent centrifugation at 10,000 rpm for 5 min to separate the clear supernatant. The spectrophotometer was used to measure the absorbance of the samples at specific wavelengths: 645 nm, 663 nm, and 480 nm. An 80 % acetone solution served as the reference during spectrophotometric analysis. The concentrations of chl a, b, total chl, and carotenoids were quantified utilizing the formula provided by [29] respectively; was followed with minor modifications [30].

### 2.5 Assessment of electrolyte leakage, hydrogen peroxide and malondialdehyde

Dionisio-Sese and Tobita [31] described a protocol to measure the extent of electrolyte leakage (EL). Initially, the leaves were chopped into tiny pieces and put in test tubes with 8 mL of distilled water. After being immersed in water for 2 h, these samples were autoclaved at 121 °C for 20 min to establish the initial electrical conductivity (EC1). Subsequently, the samples were cooled to 25 °C to determine the final electrical conductivity (EC2). EL was evaluated using either a conductivity meter (model 720, INCO-LAB Company, Kuwait) or a pH meter. After that, the EL was calculated using the given formula.

EL = (EC1 / EC2) × 100

The measurement of hydrogen peroxide (H_2_O_2_) and malondialdehyde (MDA) levels in *L. minor* leaves followed the methodologies specified by Heath and Packer [32], with modifications suggested by [[33], [34], [35]].[

### 2.6 Determination of soluble protein content, soil-plant analysis development value and antioxidant enzymes activities

The soluble protein (SP) content was assessed following Bradford's method [36], utilizing albumin as the standard and Coomassie Brilliant Blue G-250 as the dye. Soil-plant analysis development (SPAD) was measured using a SPAD-502 m sourced from Zheijang Top Instruments Co., Ltd., China.

Different procedures were used to assess antioxidant enzymes involving catalase (CAT), peroxidase (POD), superoxide dismutase (SOD), and ascorbate peroxidase (APX). Fresh leaves (0.5 g) were homogenized in liquid nitrogen, followed by 5 mL of 50 mmol sodium phosphate buffer (pH 7.0) containing 0.5 mmol EDTA and 0.15 mol NaCl. After centrifugation at 12,000×*g* for 10 min at 4 °C, the resulting supernatant was used for assessing SOD and POD activities. SOD activity was measured in a 3 mL reaction mixture consisting of 50 mM sodium phosphate buffer (pH 7), 56 mM nitro blue tetrazolium, 1.17 mM riboflavin, 10 mM methionine, and 100 μL enzyme extract. The absorbance was measured using a spectrophotometer (xMark™ Microplate Absorbance Spectrophotometer; BioRad). Enzyme activity was measured following the methodology of Chen and Pan [37] and reported as U g^−1^ FW.

The POD activity in the leaves was assessed using the methodology published by Sakharov and Ardila [38], with guaiacol as the substrate. A reaction mixture of 3 mL was prepared, which consisted of 0.05 mL of enzyme extract, 2.75 mL of 50 mM phosphate buffer (pH 7.0), 0.1 mL of 1 % H_2_O_2_, and 0.1 mL of 4 % guaiacol solution. The change in absorbance at 470 nm, resulting from the oxidation of guaiacol, was observed for 2 min. The observed absorbance values were used to calculate the enzyme activity. One unit of enzyme activity was set as the amount of enzyme required to catalyze the specified reaction.

The assessment of CAT activity adhered to the methodology followed by Aebi [39]. The experimental mixture consisted of 3.0 mL of 100 μL enzyme extract, 100 μL of H_2_O_2_ (300 mM), and 2.8 mL of 50 mM phosphate buffer with 2 mM EDTA (pH 7.0). The reduction in absorbance at 240 nm, attributed to the breakdown of H_2_O_2_ (with an extinction coefficient of 39.4 mM^−1^ cm^−1^), served as the measure of CAT activity. The quantification of APX activity followed the protocol delineated by [40]. The assay mixture consisted of 100 μL of enzyme extract, 100 μL of ascorbate (7.5 mM), 100 μL of H_2_O_2_ (300 mM), and 2.7 mL of 25 mM potassium phosphate buffer having 2 mM EDTA (pH 7.0). APX activity was assessed by monitoring the oxidation of ascorbate, with changes in absorbance at 290 nm serving as the indicator (which has an extinction coefficient of 2.8 mM^−1^ cm^−1^).

### 2.7 Determination of the chromium content

Dried and powdered samples weighing 1g of *L. minor* plants were placed into digestion flasks. 20 mL of concentrated nitric acid was introduced into each flask, and the amalgamation was subjected to heating for 20 min, gradually escalating to a temperature of 250 °C within a chemical fume hood on a hot plate. After the cooling phase, 10 mL of hydrogen perchloric acid was added to each flask. The solution was then heated again until it became colorless. After cooling, the solutions were filtered through the Whatman No. 2 filter paper. The ultimate volume of each solution was adjusted to 100 mL employing distilled water, adhering to the protocol delineated by [41]. Subsequently, these digested solutions were meticulously transferred into sterilized plastic bottles and stored at room temperature for further analytical procedures. The quantification of Cr within the digested solutions was conducted by Atomic Absorption Spectrophotometer (AAS) (Model: Savant AA, Australia).

### 2.8 Statistical analysis

Data obtained from all the treatments of experiment was presented in tabular form and figures as an average of three replicates analyzed through Statistics 8.1. Factorial Analysis of Variance (ANOVA) was employed to compare independent variables. Subsequent to this, all pairwise comparisons across different time points within treatments were examined using Tukey HSD test at a significance level of α = 0.05. Lowercase letters denote mean differences, indicating significant distinctions between values at *P ≤ 0.05.*

## 3 Results

### 3.1 Growth characteristics of *L. minor*

**Fig-1:**
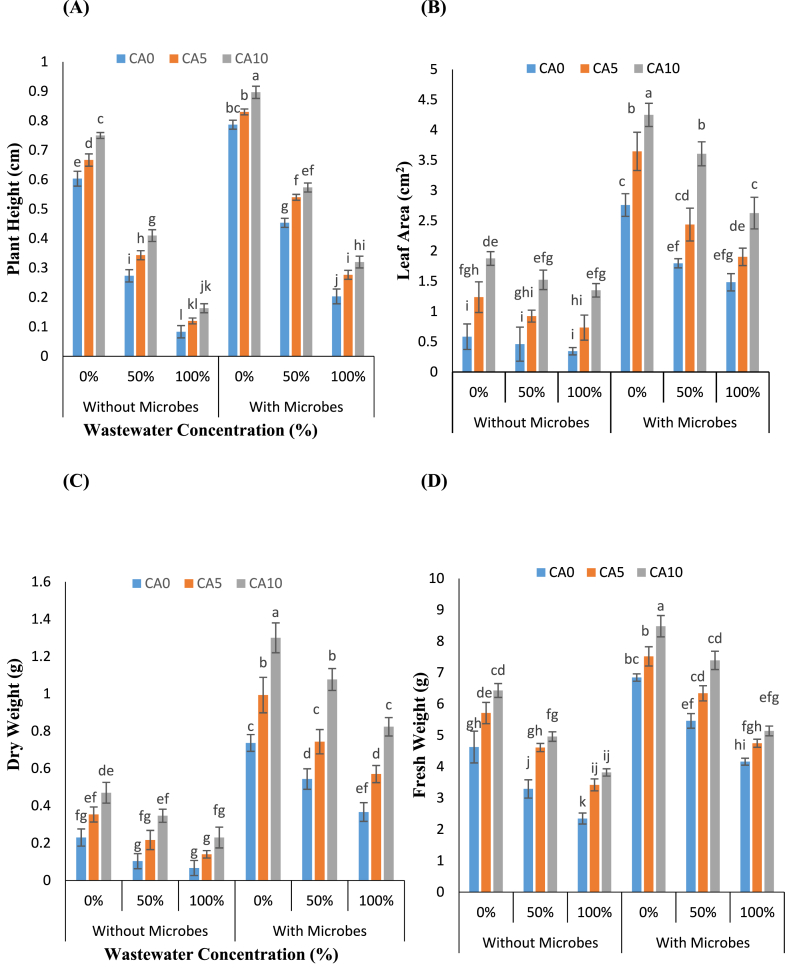
(A–D): Effects of tannery wastewater (0, 50, and 100 %), citric acid (5 and 10 mM) and *Staphylococcus aureus* on Plant height, Leaf area, Fresh and Dry weight in *Lemna minor* grown in CWs. Values are the means of three replicates along with standard deviation. Different normal or italicized small and capital letters indicate that values are significantly different at P < 0.05.

The study evaluated various growth parameters of *L. minor* to assess the impact of Cr-treated WW under the presence of single application of CA and combined application of CA and microbes. It was observed that as the levels of WW stress increased, there was a decrease in growth parameters (Fig. 1A–D). The most significant reduction was found in plants exposed to 100 % WW concentration, both in plants treated with and without microbes, when compared to the control. Specifically, plant height decreased by 86 % and 74 %, FW by 41 % and 39 %, DW by 51 % and 37 %, and leaf area decreased by 28 % and 38 %, respectively. Furthermore, the addition of CA contributed to the enhancement of plant height and leaf area under WW stress conditions. At CA concentrations of 5 and 10 mM without microbes under WW, plant height increased by 25–44 % and 50–96 %, respectively, FW by 40–63 %, DW by 103–245 %, while leaf area increased by 100–113 % and 231–293 % compared to CA (0 mM). In plants treated with microbes, CA addition (5 and 10 mM) similarly enhanced plant height by 19–36 % and 26–57 %, FW by 14–35 %, DW by 36–124 %, and leaf area by 35-28 % and 77–100 %, respectively. Overall, as wastewater concentration increased (i.e., 50 % and 100 %), there was a notable reduction in plant growth. However, the combined application of *S. aureus* and CA effectively increased the growth parameters of the tested plants, particularly mitigating the adverse effects of 100 % WW content.

### 3.2 Biochemical characteristics

**Fig-2:**
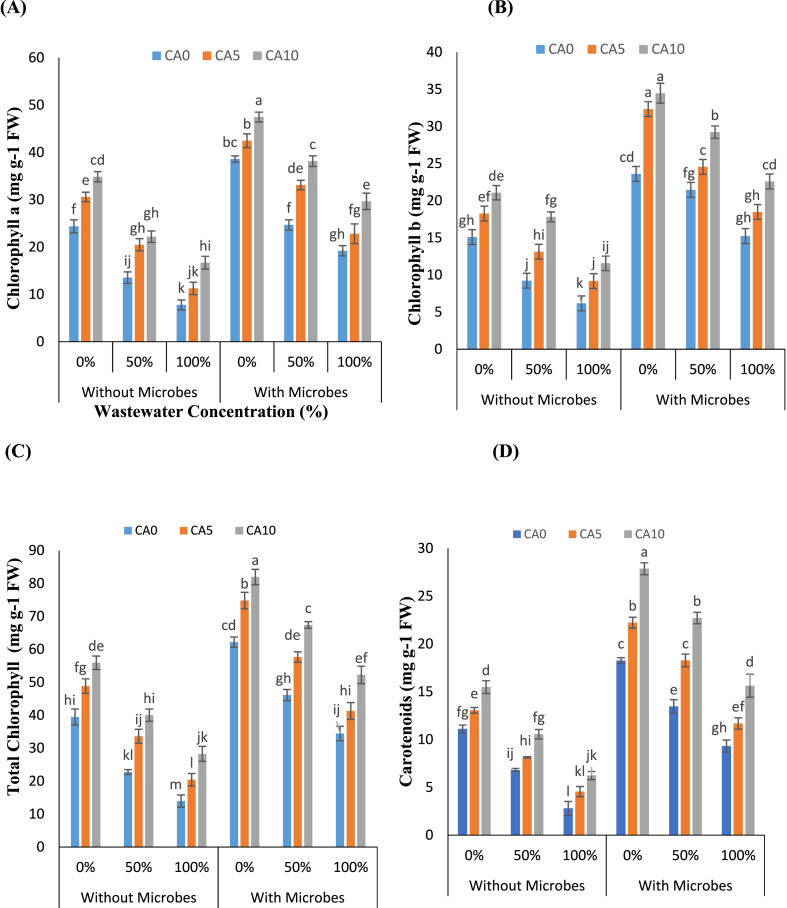
(A–D): Effects of tannery wastewater (0, 50, and 100 %), citric acid (5 and 10 mM) and *Staphylococcus aureus* on chlorophyll *a*, *b*, total chlorophyll and carotenoids in *Lemna minor* grown in CWs. Values are the means of three replicates along with standard deviation. Different normal or italicized small and capital letters indicate that values are significantly different at P < 0.05.

The foliar chlorophyll *a*, *b*, and carotenoid contents in test plants exhibited a reduction with increased uptake of WW (Fig. 2A–D). However, CA application increased chl. and carotenoid contents, thereby supporting photosynthetic activities. The most significant decline in chl content was noted at a WW concentration of 100 % compared to controls, both in plants treated with microbes and those left untreated. Specifically, the reduction in chl a was 68 % and 50 %, chl b was 59 % and 35 %, and total chl was 64 % and 44 %, respectively. In contrast, CA supplementation led to enhancements in chlorophylls content. At a concentration of 5 and 10 mM, CA increased chl a by 63–114 %, chl b by 87–93 %, and total chl by 75–102 % under wastewater stress conditions. Furthermore, in plants treated with microbes, CA (5 and 10 mM) addition similarly boosted photosynthetic pigments in the range of chl a increased by 54.60–54.38 %, chl b by 36–48 %, and total chl by 46–51 % under 100 % WW stress with CA (5 and 10 mM) in combination without microbes. Overall, this research outlined that the exogenous treatment of CA in conjunction with *S. aureus* significantly increased carotenoid and chlorophyll (a, b, and total) contents.

### 3.3 SP content and SPAD value

**Fig-3:**
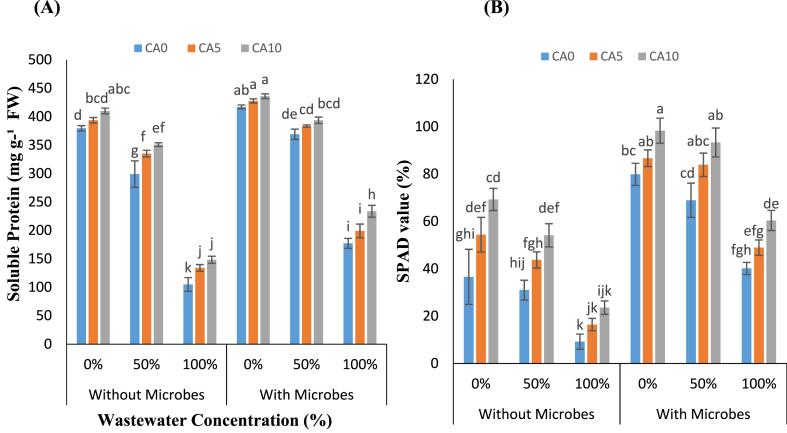
(A–B): Effects of tannery wastewater (0, 50, and 100 %), citric acid (5 and 10 mM) and *Staphylococcus aureus* on Soluble protein, and Soil plant analysis develoment in *Lemna minor* grown in CWs. Values are the means of three replicates along with standard deviation. Different normal or italicized small and capital letters indicate that values are significantly different at P < 0.05.

The SP content of *L. minor* exhibited a decrease with increasing concentrations of WW. Notably, lants treated without microbes displayed the lowest SP values under increased WW concentrations, particularly at 100 % (Fig. 3A and B). Conversely, the highest SP values were noted in plants treated with microbes and CA at a concentration of 10 mM, compared to the 100 % WW only. Additionally, the SPAD value decreased in all plants with increased levels of WW. The lowest SPAD values were recorded at the highest wastewater concentration (100 %). Conversely, the highest SPAD values were observed with the combined application of CA (10 mM) and microbes, compared to the 100 % WW.

Moreover, significant reductions were observed under wastewater concentration (100 %) compared to controls in plants without microbial treatment, with reductions of 72 % for SP, and 74 % for SPAD, respectively. However, CA treatments (5 and 10 mM) without microbes resulted in improved SP and SPAD values, showing increases in the range of 12–27 % and 17–41 % under WW stress. Interestingly, similar improvements in SP and SPAD values were observed in *L. minor* when CA was applied in conjunction with microbes under WW conditions. In plants treated with microbes, the addition of CA increased the SP and SPAD values, with improvements in the range of 3–12 % for SP and 6–31 % for SPAD at CA concentrations of 5 and 10 mM, respectively. Thus, the combined treatment of CA with microbes significantly enhanced SP content and SPAD values in *L. minor,* demonstrating its potential to mitigate the adverse effects of wastewater stress.

### 3.4 Antioxidant enzyme activities

**Fig-4:**
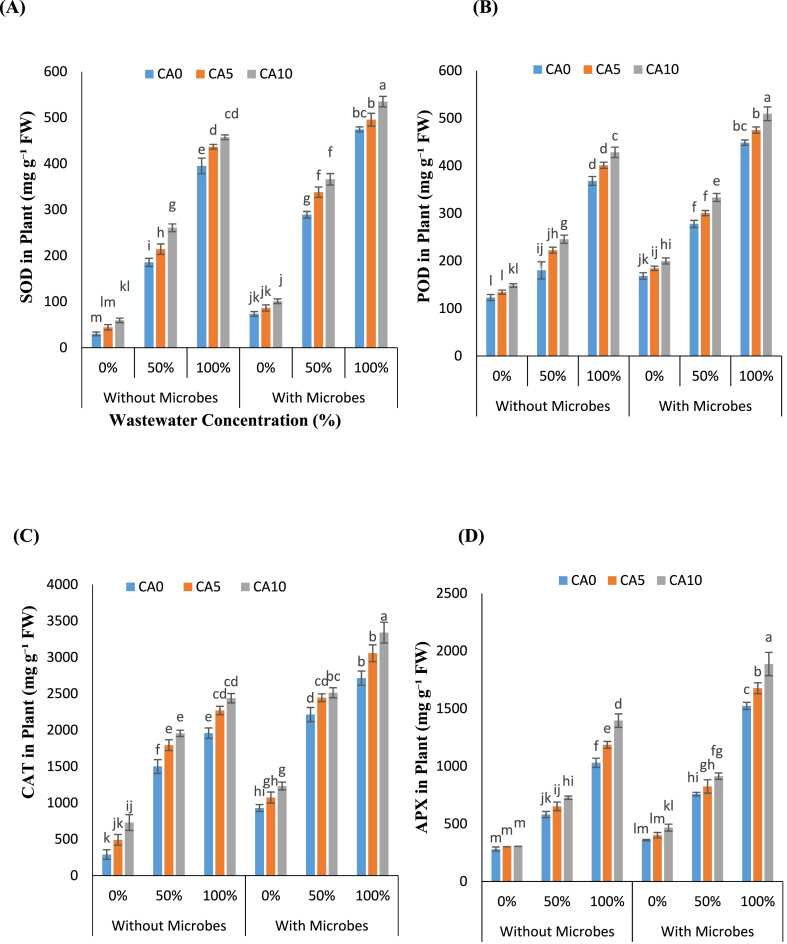
(A–D): Effects of tannery wastewater (0, 50, and 100 %), citric acid (5 and 10 mM) and *Staphylococcus aureus* on SOD, POD, CAT and APX in *Lemna minor* grown in CWs. Values are the means of three replicates along with standard deviation. Different normal or italicized small and capital letters indicate that values are significantly different at P < 0.05.

The study investigated the antioxidant activities, including APX, CAT, POD, and SOD, to evaluate the defense mechanisms against oxidative damage in *L. minor*. The presence of Cr during plant uptake was found to induce stress, leading to increased production of oxidants (Fig. 4A–D). Notably, the lowest levels of antioxidant enzymes were observed in plants exposed to 0 % WW levels. Conversely, plants devoid of microbes exhibited the highest levels of antioxidant enzymes when subjected to 100 % WW levels, especially in conjunction with the chelating agent (CA) at a concentration of 10 mM, compared to 0 %.

However, the addition of CA (5 and 10 mM) without microbes increased SOD by 10–15 % and 15–40 %, POD by 8–23 % and 16–36 %, APX by 12–15 % and 25–35 %, and CAT by 15–19 % and 24–30 %, respectively. Interestingly, CA supplementation (5 and 10 mM) with microbes resulted double increase in same doses of CA (5 and 10 mM) without microbes in SOD by 4–16 % and 12–26 %, POD by 5–8% and 13–20 %, APX by 8–10 % and 20–23 %, and CAT by 10–12 % and 13–23 % at CA concentrations of 5 and 10 mM, respectively. These findings underscore the potential of CA, particularly in combination with microbial treatment, to enhance the antioxidant defense system in *L. minor* under conditions of WW stress, thus aiding in the mitigation of oxidative damage.

### 3.5 Oxidative stress in *L. minor*

**Fig-5:**
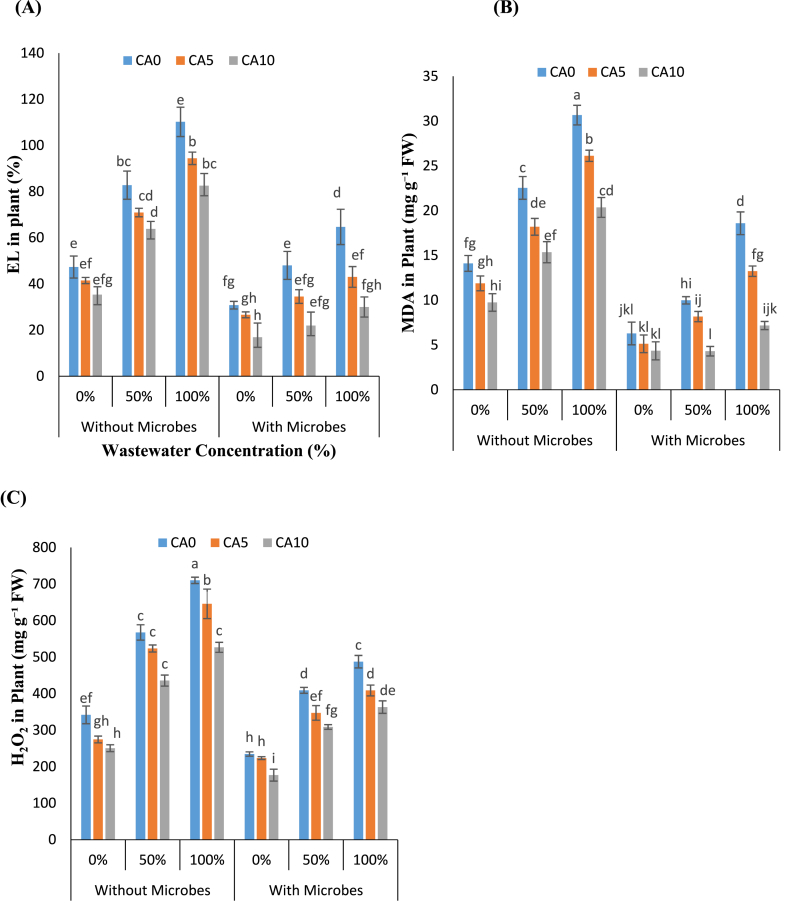
(A–C): Effects of tannery wastewater (0, 50, and 100 %), citric acid (5 and 10 mM) and *Staphylococcus aureus* on EL, MDA and H_2_O_2_ in *Lemna minor* grown in CWs. Values are the means of three replicates along with standard deviation. Different normal or italicized small and capital letters indicate that values are significantly different at P < 0.05.

The assessment of oxidative damage in *L. minor* was conducted by measuring parameters such as EL, MDA, and H_2_O_2_ in response to varying concentrations of wastewater. The results revealed a notable increase in EL, MDA, and H_2_O_2_ levels corresponding to higher concentrations of WW (Fig. 5A–C). Interestingly, treatments involving the application of a chelating agent (CA) at concentrations of 10 mM, particularly in conjunction with *S. aureus*, demonstrated the most significant reduction in MDA, EL, and H_2_O_2_ at 100 % WW levels compared to the same WW without CA and *S. aureus*. Specifically, plants treated with CA without microbial exhibited the lowest values across all measured parameters for EL 14.32–14.34 % & 22–25 %, MDA 14–19 % & 31–33 %, H₂O₂ 7–9% & 23–25 %, respectively.

Specifically, CA (5 and 10 mM) with *S. aureus* exhibited a remarkable decrease in EL 28–33 % & 53–54 %, MDA 18–28 % & 56–61 %, H₂O₂ 15–16 % & 24–25 % in the presence of WW (50 and 100 %). Microbes and CA, however, significantly reduced EL, MDA, and H_2_O_2_ within plants, highlighting their potential to mitigate oxidative stress. Overall, the findings underscore the importance of exogenously applying Cr-resistant bacteria with CA to effectively suppress oxidative stress indicators in *L. minor* subjected to WW contamination.

### 3.6 Chromium concentration and accumulation in *L. minor*

The extent of plant uptake was contingent upon the percentage of WW concentration, with the highest metal accumulation observed at 100 % wastewater concentration, particularly in plants treated with both microbes and CA (10 mM) (Table 1). Specifically, Cr concentration displayed a notable increase of 147 % in plants without microbial treatment and 149 % in those with microbial treatment when exposed to 100 % wastewater compared to 50 % WW.

**Table 1:** Effects of Cr present in tannery wastewater (0, 50 and 100 %) with and without microbes in combination with CA (5 and 10 mM) on chromium concentration and accumulation in *L. minor*.

Treatments	Cr Concentration (mg kg^−1^)	Cr accumulation (μg plant^−1^)	Treatments	Cr Concentration (mg kg^−1^)	Cr accumulation (μg plant^−1^)
T.E 0 % + CA (0 mM)	0.17 ± 0.01l	0.03 ± 0.01f	T.E 0 % + CA (0 mM) + M	0.33 ± 0.01l	0.24 ± 0.02f
T.E 0 % + CA (5 mM)	0.21 ± 0.01l	0.07 ± 0.01f	T.E 0 % + CA (5 mM) + M	0.43 ± 0.01l	0.42 ± 0.05f
T.E 0 % + CA (10 mM)	0.26 ± 0.01l	0.12 ± 0.01f	T.E 0 % + CA (10 mM) + M	0.59 ± 0.02l	0.76 ± 0.07f
T.E 50 % + CA (0 mM)	125.36 ± 5.02k	13.04 ± 5.32ef	T.E 50 % + CA (0 mM) + M	170.1 ± 1.02i	92.45 ± 9.92de
T.E 50 % + CA (5 mM)	145.25 ± 1.29j	31.51 ± 7.70ef	T.E 50 % + CA (5 mM) + M	209.48 ± 1.11h	155.76 ± 14.4de
T.E 50 % + CA (10 mM)	164.53 ± 3.55i	57.12 ± 6.99ef	T.E 50 % + CA (10 mM) + M	239.94 ± 1.33g	258.28 ± 12.66d
T.E 100 % + CA (0 mM)	310.34 ± 8.94f	20.92 ± 13.18d	T.E 100 % + CA (0 mM) + M	423.60 ± 7.85c	155.48 ± 23.48b
T.E 100 % + CA (5 mM)	349.64 ± 6.40e	49.01 ± 7.77c	T.E 100 % + CA (5 mM) + M	478.39 ± 16.5b	273.18 ± 31.09b
T.E 100 % + CA (10 mM)	383.30 ± 7.34d	88.33 ± 22.71c	T.E 100 % + CA (10 mM) + M	538.38 ± 7.73a	443.49 ± 32.59a

The CA at concentrations (5 and 10 mM) increased the concentration and accumulation of Cr in L. *minor.* Specifically, CA application resulted in an enhancement ranging from 12 % to 15 % and 23 %–31 % in concentration, and 134 %–141 % and 322 %–337 % in accumulation, respectively. Moreover, when combined with *S. aureus* treatment, CA application (5 and 10 mM) further bolstered the concentration and accumulation of Cr in L. *minor*. The enhancement ranged from 12 % to 23 % and 27 %–41 % in concentration, 68 %–75 %, and 179 %–185 % in accumulation, respectively. Thus, the results emphasize the significance of applying Cr-resistant bacteria in conjunction with CA to effectively augment Cr uptake in *L. minor* exposed to tannery effluents.

## 4 Discussion

Chromium (Cr) alters its oxidation states due to its high redox potential. At high levels, Cr is a threat to the biological structural integrity by disrupting membranes, lipids, proteins, and DNA. The movement of Cr within plants is influenced by its concentration, oxidation state, and the particular plant species it encounters [42]. Cr is widely recognized as a hazardous metal capable of impairing the growth and development of plants [[22], [43]]. Numerous studies have reported its harmful effects on morphology and biochemistry, including plant germination, root growth, stem development, leaf development, and photosynthetic efficiency across multiple plant species [[44], [45]]. Alghanem et al [46] conducted a study that revealed that exposure to tannery effluent led to a decrease in the chlorophyll and protein content in submerged aquatic plants. Similarly [47], observed diminished growth parameters and photosynthetic pigments in *Spirodela polyrrhiza* following exposure to such effluent. *Callitriche cophocarpa* has efficiency in accumulating Cr and maintaining physiological conditions even under high Cr concentrations. Various plants show reduced photosynthetic functionality and efficiency due to high levels of HMs and including Cr, which can inhibit chlorophyll biosynthesis, functionality of photosystems and altering chlorophyll levels [[48], [49], [50]][].

Our research showed that Cr toxicity lowered chlorophyll contents, which in turn affected photosynthesis and resulted in reduced plant growth. However, CA improved these parameters and positively correlated with *L. minor* experiencing WW stress (Fig. 2A–D). Mahdavian [51] demonstrated that CA supplementation enhanced the antioxidant activity of garden cress under Cr stress conditions, thereby improving morphological and physiological characteristics. Ilyas et al. [7] found that treating plants with CA mitigated the toxic effects of Cr and increased antioxidant enzyme activities. CA holds potential as a means to counteract the adverse effects of Cr on plant structure and metabolism. This study examines the positive correlation between *S. aureus* and CA in wastewater, which helps *L. minor* cope with WW-induced stress. Congeevaram et al. [52] suggests that bacteria and fungi, including *Micrococcus* sp. and *Aspergillus* sp., can reduce the impact of Cr by extracting it from industrial WW. This notion finds support in the work of Ahmad et al. [25], who delves into the interactions between Cr and bacteria, suggesting their potential role in mitigating the metal's toxic effects. Hence, it is conceivable that bacteria may contribute to alleviating the impact of Cr on macrophytes.

As the level of WW stress increased, there was a noticeable increase in the formation of MDA and EL (Fig. 5A–C). This suggested potential damage or instability in the cell membrane, initiating oxidative stress characterized by the production of H_2_O_2_ and superoxide radicals (O^2−^). The overproduction of ROS like H_2_O_2_ and O^2−^ can result in the oxidation of proteins, peroxidation of lipids, disruption of DNA and RNA strands, and inhibition of enzyme activities [53]. The excess ROS induced by Cr metal triggers the formation of MDA, ultimately leading to the breakdown of fatty acids within cell membranes [54]. Under conditions of Cr stress, there was a significant increase in H_2_O_2_ generation coupled with elevated MDA formation in *L. minor* plants, which is consistent with the findings of Ishaq et al. [18]. The fact that Cr induces oxidative stress and MDA production strongly indicates the harmful effects on cells. This study explains that CA effectively reduces ROS and oxidative stress in plants exposed to WW stress. ROS, such as H_2_O_2_ and O^2−^, are naturally generated within plant cells as byproducts of metabolic processes; however, their excessive accumulation can result in oxidative damage and cell demise [54]. CA reduces plant cell oxidative stress by scavenging ROS using its natural antioxidant properties [55].

Moreover, the potential of beneficial microbes to mitigate ROS production and levels in irrigated cropping systems, thereby alleviating oxidative stress, has been extensively documented. Sahu et al. [56] further underscores the pivotal role of endophytic microbes in maintaining ROS homeostasis in plants under environmental stress. Oxidative stress-tolerant microalgae strain capable of thriving in WW exhibit high APX activity for ROS scavenging. Additionally, Janani et al. [57] has demonstrated that *Daphnia magna*, a macrophyte, can effectively cope with ROS present in treated effluents, and also suggesting a potential role for *S. aureus* in mitigating oxidative stress in macrophytes under wastewater conditions. Our experiment is consistent with previous studies and highlight the significant role of microbes in reducing oxidative stress and ROS levels in *L. minor* exposed to WW conditions (Fig. 5A–C).

The accumulation of ROS minimizes by various antioxidant enzymes, including SOD, POD, APX, and CAT. Multiple studies demonstrate that these antioxidative enzymes reduce Cr toxicity in various plant species. This response is believed to be a defense mechanism against the oxidative stress induced by Cr [58]. However, elevated concentrations of Cr present in tannery effluent can hinder plant growth and associated enzymatic activities. Consequently, CA to enhance the activities of antioxidant enzymes in *L. minor* under WW stress represents a promising strategy to enhance their resilience to environmental stressors (Fig. 4A–D). Moreover, the supplementation of chelating agents has been demonstrated to alleviate Cr toxicity in spinach plants, resulting in augmented activities of antioxidative enzymes Mahdavian, 2021.

Microbes have emerged as beneficial agents in enhancing the activities of antioxidant enzymes within plants. These enzymes play a pivotal role in safeguarding plants against oxidative stress induced by a myriad of biotic and abiotic factors [59,60]. The synergistic action of rhizosphere microbes significantly augmented the activities of CAT, glutathione reductase (GR), and guaiacol peroxidase (GPx) in chickpea plants, thereby bolstering defense mechanisms against pathogenic threats [61]. Similarly, documented those treatments involving plant activators and microbial interventions resulted in heightened antioxidant activities in tomatoes. This augmentation in enzyme activities contributed to a reduction in disease severity and the restoration of photosynthetic balance. Thus, this study highlights the significance of increased enzyme levels in *L. minor* through the combined application of citric acid and *S. aureus.* This synergy suggests a relationship that provides protection against oxidative stress and enables adaptation to Cr-stressed environments.

HMs in water bodies can create significant environmental challenges that can reduce plants' SP levels and SPAD content. Our analyses indicated that WW stress lowered the SPAD and SP levels in plant tissues (Fig. 3A and B). These declines in SP levels were attributed to the enrichment of heavy metals in plant tissues, thereby enhancing the formation of ROS. The impact of HM stress on gas exchange characteristics is profound in *Cynodon dactylon* and *Cenchrus ciliaris* under Cd stress [62]. Organic acids with wastewater can improve plants’ growth, photosynthetic efficiency, and gas exchange. Additionally, fertilizer application of oxalic acid has been found to significantly increase plant height and leaf SPAD value in *Sorghum bicolor* plants compared to unfertilized control plants [63]. Furthermore, amino acid chelators increased SPAD values and SP levels while reducing ROS and EL production. Salicylic and ascorbic acid enhance plant responses and water use efficiency [64]. The exogenous citric acid induces increased soluble protein levels and SPAD values in *L. minor.*

Our study suggested that SP levels and SPAD values were more pronounced in *L. minor* when treated with *S. aureus* and CA. Furthermore, plant growth-promoting rhizobacteria (PGPR) aids in mitigating metal stress in halophytes by reducing the activity and gene expression of ROS-scavenging enzymes [65]. Furthermore, research has investigated the role of bacterial extracellular polymeric substances (EPS) in detoxifying various HMs. The production of EPS is induced by toxic metals, enhancing the adsorption and detoxification capacity of the bacteria [66]. These findings highlighted the potential of microbial treatments and CA supplementation in improving protein content, which may exhibit resilience to metal stress in *L.* *minor*.

Interestingly, this investigation revealed that chromium-resistant *S. aureus* K1 may possess the capability to reduce Cr^6+^ to Cr^3+^. However, the precise mechanism underlying this Cr reduction from (VI) to (III) form remains unidentified. Previous studies suggested that metal reduction may occur through intracellular metabolism, where chromate acts as an electron acceptor to produce energy and facilitate Cr^6+^ detoxification [67]. Additionally, metabolic byproducts such as H_2_S and enzymatic activities of bacteria contribute to Cr^6+^ to Cr^3+^ [25,8]. Numerous studies have identified various bacteria, including *Streptomyces griseus* [68]*, Rhodococcus erythropolis* [69]*, Bacillus pumilus, Alcaligenes faecalis*, and *Staphylococcus* sp. [67] as well as *Bacillus cereus* (Moreno-Benavides, 2019), capable of mediating the reduction of Cr^6+^ to Cr^3+^ in WW. There are reports that bacteria can convert Cr^6+^ concentrations within 24–48 h [68]. Microbial agents show promise in the bioremediation of Cr^6+^-contaminated WW, reducing it to regulatory-compliant levels [[70], [71]].

*Lemna minor* has been demonstrated as an effective agent for Cr removal from WW, exhibiting increased bio uptake capacity with elevated temperatures and decreased initial Cr concentrations Ishaq et al., 2021. Additionally, the supplementation of CA has been identified as a means to augment Cr uptake by *Lemna minor*, thereby enhancing its phytoextraction potential [72]. Supporting these findings, Uysal (2013) provides further evidence of *Lemna minor's* proficiency in Cr ion removal from WW, with maximal accumulation observed in plants cultivated in a pond environment characterized by a pH of 4.0 and an initial Cr concentration of 5.0 mg/L. Our research is in line with these studies and found that *L. minor* can significantly reduce Cr concentration in tannery WW (Table 1).

The study highlighted the positive effects of supplementing CA and *S. aureus* K1 on various physiological attributes, antioxidant enzymes, and soluble proteins of *L. minor* under WW stress conditions. These results pointed out the potential of employing phytoremediation (i.e. *L. minor*) coupled with biotechnological interventions, such as microbial augmentation (*S. aureus*) and chelating agent (i.e. CA) application, as promising avenues for developing environmentally friendly and sustainable solutions for chromium removal from wastewater. *Lemna minor* emerges as a promising candidate for Cr removal, while the involvement of *S. aureus* proveed instrumental in Cr level transformation, suggesting its potential inclusion in tannery effluents. Strategically placing *L.* *minor* near tanneries can increase effectiveness. Additionally, CA demonstrated significant utility in bolstering the resilience of *L. minor* under WW stressed conditions.

## 5 Conclusion

The study primarily focused on assessing the efficacy of *Lemna minor* in Cr remediation, particularly in the presence of citric acid and microbes. The application of citric acid with *Staphylococcus aureus* strain K1 improved the morpho-physiological characteristics of *L. minor* under the exposure to the polluted wastewater. It was observed that escalating the levels of WW containing Cr, intensified the oxidative stress in test plants. The combined application of CA and *S. aureus* enhanced the antioxidant activities in test plants and ameliorated the oxidative stress. Interestingly, *L. minor* demonstrated biological competence in absorbing Cr from industrial effluents. The presence of *S. aureus* positively influenced the conversion of Cr into a form that was more readily accessible to *L. minor,* thereby enhancing its accumulation. Moreover, CA supplementation further facilitated Cr accumulation in *L. minor.* Consequently, the concurrent application of CA and *S. aureus* proved highly effective in promoting Cr accumulation. Employing a combination of *S. aureus* with *L. minor* holds promise to deliver more efficient remediation of industrial effluents. However, it's imperative to acknowledge that while this study sheds light on the efficacy of *Lemna minor*-mediated phytoremediation combined with biotechnological interventions, further research employing different species is warranted to validate this technique on a broader scale. This study suggested combining phytoremediation, microbial augmentation, and chelating agent application could be a promising approach for treating Cr-toxic wastewater. Moving forward further research is needed to determine its effectiveness and feasibility in different environmental conditions.

## Ethics approval

Not applicable.

## Consent to Participate

Not applicable.

## Consent to publish

The authors give their consent to publish.

## Funding

This project was funded by the Higher Education Commission (HEC) of Pakistan under National Research Program for Universities (NRPU) grant No. HEC/R&D/NRPU/2017/8996. The authors also expressed their gratitute to the 10.13039/501100004242Princess Nourah bint Abdulrahman University Researchers Supporting Project number (PNURSP2024R93), Princess Nourah bint Abdulrahman University, Riyadh, Saudi Arabia.

## Availability of data and materials

This will be available on request.

## CRediT authorship contribution statement

**Rahat Arshad:** Writing – original draft, Methodology, Investigation, Formal analysis, Data curation. **Arwa Abdulkreem AL-Huqail:** Writing – review & editing, Visualization, Funding acquisition. **Suliman Mohammed Suliman Alghanem:** Writing – review & editing. **Ibtisam Mohammed Alsudays:** Writing – review & editing. **Mujahid Farid:** Writing – review & editing, Visualization, Project administration, Investigation, Funding acquisition, Conceptualization. **Wajiha Sarfraz:** Writing – review & editing, Writing – original draft, Validation, Methodology, Formal analysis. **Mohsin Abbas:** Visualization, Software, Resources. **Zaki ul Zaman Asam:** Visualization, Resources. **Noreen Khalid:** Writing – review & editing. **Jean Wan Hong Yong:** Writing – review & editing, Funding acquisition, Formal analysis, Data curation. **Amany H.A. Abeed:** Writing – review & editing.

## Declaration of competing interest

The authors declared that they have no known competing financial interests or personal relationships that could have appeared to influence the work reported in this paper.
