# Supplementary material for: Phyto-treatment of tannery industry effluents under combined application of citric acid and chromium-reducing bacterial strain through Lemna minor L.: A lab scale study
Source: Heliyon. 2024 Aug 13;10(16):e36309. doi: 10.1016/j.heliyon.2024.e36309 (PMC11382062; doi:10.1016/j.heliyon.2024.e36309)
Supplement: Multimedia component 1 [file mmc1.docx]

**Supplementary Table 1: Physicochemical characterization of wastewater sample from tannery industry.**

| Tannery Effluent Properties | | Tannery Effluent Properties | |
| --- | --- | --- | --- |
| Parameter | **Concentration** | **Parameter** | **Concentration** |
| Temperature (In Lab) | 23.6 °C | Total Suspended Solids | 18 mg/L |
| pH value | 8.1 | Arsenic (As) | 1.4 mg/L |
| Conductivity | 3140 μS/*cm* | Barium (Ba) | 1.9 mg/L |
| Total dissolved solids (TDS) | 1727 mg/L | Boron (B) | 7.2 mg/L |
| Suplphate | 66 mg/L | Cadmium (Cd) | 3 mg/L |
| Flouride | 3.28 mg/L | Chromium (Cr) | 3.8 mg/L |
| Total Chlorine | 2.3 mg/L | Copper (Cu) | 2.1 mg/L |
| Cyanide | 2.7 mg/L | Iron (Fe) | 9.2 mg/L |
| Total Hardness (As OACO_3_) | 364 mg/L | Lead (Pb) | 1.3 mg/L |
| Chloride | 308 mg/L | Manganese (Mn) | 1.8 mg/L |
| Sulfide | 1.8 mg/L | Mercury (Hg) | 1 mg/L |
| Oil and Grease | 8.23 mg/L | Nickle (Ni) | 0.1 mg/L |
| Chemical Oxygen Demand (COD) | 267 mg/L | Silver (Ag) | 1.2 mg/L |
| Biochemical Oxygen Demand (BOD) | 162 mg/L | Selenium (Se) | 0.8 mg/L |
| Phenol Compounds | 1.3 mg/L | Chromium (VI) | 10 mg/L |
